# Triggering Postural Movements With Virtual Reality Technology in Healthy Young and Older Adults: A Cross-Sectional Validation Study for Early Dementia Screening

**DOI:** 10.3389/fmed.2020.533675

**Published:** 2020-11-16

**Authors:** Yu Imaoka, Nadja Saba, Anne Vanhoestenberghe, Eling D. de Bruin

**Affiliations:** ^1^Motor Control & Learning Laboratory, Institute of Human Movement Sciences and Sport, Department of Health Sciences and Technology, ETH Zurich, Zurich, Switzerland; ^2^Aspire Centre for Rehabilitation Engineering and Assistive Technologies, University College London, London, United Kingdom; ^3^Division of Physiotherapy, Department of Neurobiology, Care Sciences and Society, Karolinska Institute, Stockholm, Sweden

**Keywords:** posture, virtual reality, dementia, early diagnosis, aging, postural sway, balance assessment, head-mounted display (HMD)

## Abstract

With the ultimate aim of early diagnosis of dementia, a new body balance assessment system with integrated head-mounted display-based virtual reality (VR) has been developed. We hypothesized that people would sway more in anterior-posterior (AP) direction when they were exposed to a VR environment where we intentionally provoked movements in forward and backward directions. A total of 14 healthy older adults (OA) (73.14±4.26 years) and 15 healthy young adults (YA) (24.93±1.49 years) were assessed for group differences in sway behavior. Body sway speed in 22 different conditions with and without VR environments was analyzed. Significant differences and large effect sizes were observed in AP sway under the VR environments (OA with *P* < 0.02; effect size> 0.61, YA with *P* < 0.003; effect size> 0.72) compared to the baseline condition without the VR environments. In addition, significant differences were found between the two groups in AP sway in all test conditions (*P* < 0.01). Our study shows that a VR environment can trigger body sway in an expected direction, which may indicate that it is possible to enhance the sensitivity of balance assessment by integrating immersive VR environments. The result of this study warrants a cross-sectional study in which OA diagnosed with and without dementia are compared on their sway behavior.

## 1. Introduction

In our growing aging society, dementia is becoming a relevant problem across the globe. The deaths due to Alzheimer's disease (AD) and other dementias more than doubled between 1990 and 2016 to rank fifth among all causes of death (1, 2). In addition, the population with dementia is expected to more than triple from 2018 to 2050 to reach 152 million, and the total worldwide cost of dementia is estimated to reach US$ 2 trillion in 2030 (3). However, despite the dramatic increase in the population with dementia, there is presently no effective disease-modifying cure or treatment (2). The early diagnosis and detection of dementia disease is important, allowing people with dementia and their families more preventive intervention options. It is reported that both physical activity and cognitive intervention improve the cognition of people with cognitive impairments (4, 5). Maintaining their independent living for longer periods would lead to reducing the economic burden on the society of institutional care (6). A straightforward and objective diagnostic system using biomarkers would be beneficial for the screening and the early diagnosis and classification of dementia (2, 3, 7).

Among various potential biomarkers for dementia screening, a biomechanical approach could be useful. Previous studies confirmed that elderly people suffering from frailty were at higher risk of developing cognitive disorders compared to non-frail people (8). Interestingly, research found that the prevalence of frailty was different between dementia subtypes: AD and Lewy Bodies (9). While a systematic review observed no gold standard frailty assessment scale for primary care, assessment of postural sway could be a potential tool (10). In fact, static balance features of people with AD and mild cognitive impairments (MCI) indicate that anterior-posterior (AP) sway is a sensitive parameter to distinguish people with AD and MCI from healthy older adults (OA) (11, 12). A meta-analysis concluded that balance parameters in AP direction under the eyes-open condition were more relevant discriminators of MCI than sway in the medio-lateral (ML) direction (13). Another research investigated the association between biomechanical functions: dual-task walking and postural sway and brain activities: the functional connectivity within Default Mode Network (DMN) and between the networks of DMN and Frontoparietal Network (FPN) and of DMN and Supplementary Motor Areas (SMA) for people with MCI, using Functional Magnetic Resonance Imaging (fMRI) (14). The study revealed that the functional connectivity between DMN and SMA was significantly associated with increased postural sway in eyes-open condition, suggesting that DMN was influencing SMA possibly due to the limited attentional resources of the OA with MCI, while DMN and SMA were supposed to be deactivated and active respectively during the task in healthy older adults. The effect of a dual task on posture was evaluated for both healthy OA and people with AD, finding significant differences in people with AD between the single task (standing on a force platform) and the dual task (a cognitive task in addition to standing) (15). However, since the study focused on a declarative memory task for the cognitive load, the test required substantial time and supervision resources of both participants and experimenters. To improve the assessment system with the dual task, a passive and unsupervised assessment method would be more efficient and objective (16, 17). A dual task assessment of body balance in a more passive and unsupervised way could thus be an efficient promising discriminator of MCI if different sway behavior in the AP direction can be identified.

Virtual reality (VR) technology could help us to achieve such an assessment system and may play an important role in the diagnosis of MCI, where neuropsychological tests have been often employed in both paper-and-pencil and automated computerized formats (18, 19). For example, a review of studies comparing conventional neuropsychological assessments and VR-based assessments supports the sensitivity of VR-based assessment systems in detecting cognitive impairments and, thus, reveals the potential of VR applications for neuropsychological assessment in clinical settings (20). However, most of the current VR applications do not provide immersive VR environments with levels that are sufficient enough to provoke a balance reaction while assessing postural control in AD and MCI. A novel design is needed using more specific contexts provoking postural sway (21). Episodic memory assessments using VR are mainly based on non-immersive systems, however, they may be improved by systematically changing the degree of immersion and interactivity (22). A head-mounted display (HMD) has the potential to provide more immersive VR environments. Test-retest reliability of visual processing components revealed that HMD-based assessment was comparable to the assessment with standard CRT computer screen, thus suggesting that HMD was applicable for standardized and reliable neuropsychological assessment (23, 24).

Considering the potential of HMD-based VR technology to generate more immersive VR experience that would contribute to the dual task paradigm and improve the sensitivity of existing assessments, we aimed to develop a new theory-based body balance assessment system consisting of a stabilometer and HMD-based VR technology. To the best of our knowledge, no research has developed and evaluated a combined system for OA. In the present research, we developed a balance assessment with integrated HMD-based VR with the aim of evaluating the effect of HMD-based VR environments on body balance for healthy OA and young adults (YA). We primarily hypothesized (1) that people would sway more in the AP direction when they saw a VR scenery that was deliberately moved forward and backward than when they stood in a conventional way (with eyes-open and without a VR environment) and (2) that postural sway behavior would differ between healthy OA and YA. Along with the primary hypotheses, we had secondary hypotheses that changes of VR environments and foot position in the real world would affect postural sway of people and that the postural sway behavior might be associated with their cognitive function.

## 2. Research Method

### 2.1. Study Design

In this research, we conduct a cross-sectional study to evaluate whether a VR environment designed with specific intentions will affect postural sway in the real world and whether there will be differences in body balance parameters between healthy OA and YA. The new balance assessment system consists of a stabilometer and HMD-based VR and creates various VR designs by changing three parameters that are expected to cause different effects on measures of postural sway. Subsequently, we measure body balance of subjects under the conditions both with and without VR environments. Finally, we analyze the data statistically by comparing the test conditions between with and without VR environments and between OA and YA.

### 2.2. Preparation for Measurement

The project was organized at ETH Zurich in Switzerland from February to December, 2018. The ethics were approved by ETH Zurich Ethics Commission (registration number 2018-N-43). We recruited healthy older (over 65 years old) and young (between 18 and 35 years old) adults in Switzerland by sending leaflets describing the project overview to institutions of sports and nursing care and by publishing the recruitment information online via Seniorweb. The inclusion criteria were that the participants were physically and mentally healthy without mobility and cognitive impairments. The participants answered a health questionnaire and were tested with the Montreal Cognitive Assessment (MoCA) in advance. If severe health problems were observed, or if MoCA points were below 26, the participants were excluded.

### 2.3. Experimental Protocol

#### 2.3.1. Gait Analysis

**Figure 1:**
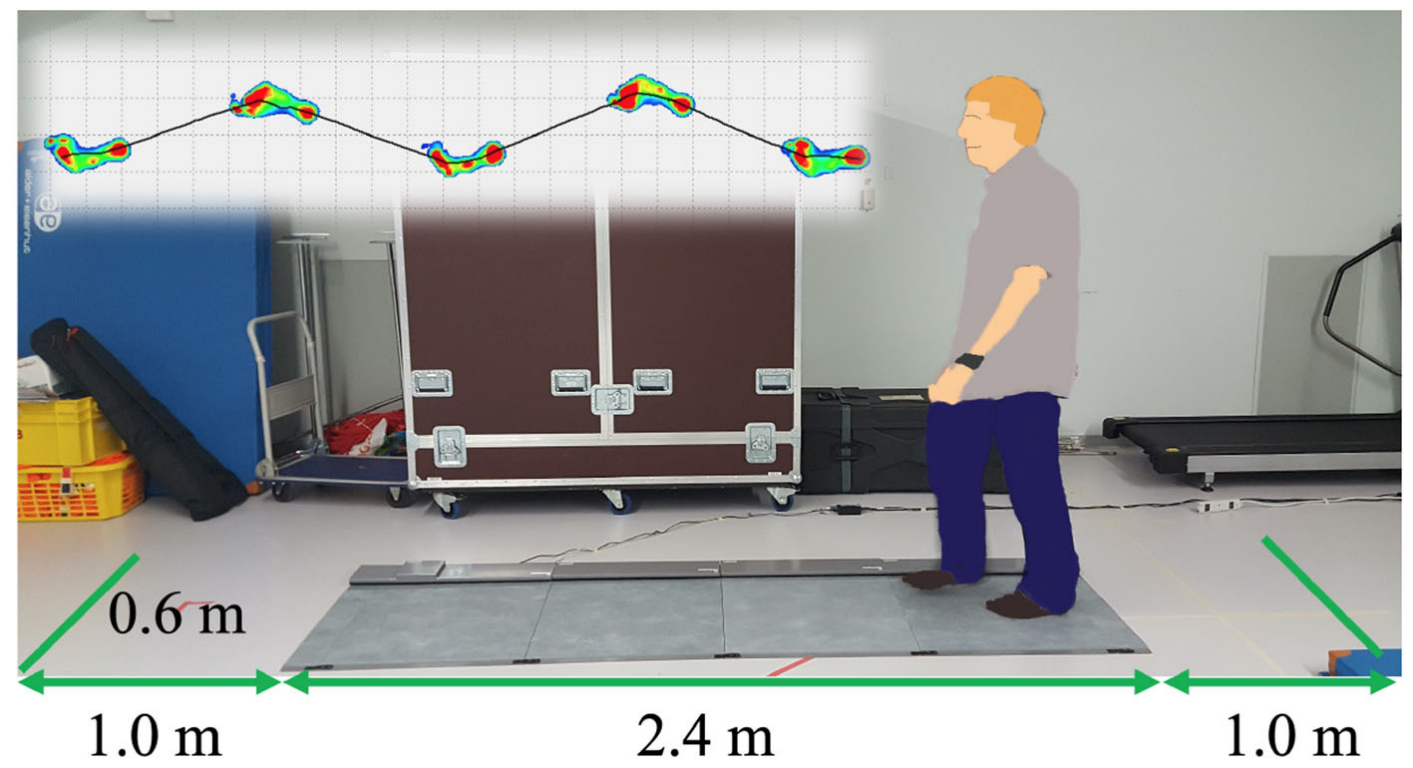
Gait analysis system with the Walkway MW-1000 (ANIMA Corp.).

At the beginning, we measured gait with a Walkway MW-1000 (ANIMA Corporation, Tokyo, Japan, Figure 1). The participants walked on the Walkway for three times, starting one meter back from the edge of the mat and finishing one meter in front of the edge. Each trial took 10 s and gait data were sampled at the frequency of 100Hz. The analysis software of the Walkway calculated the gait speed and stride length for each trial, and we averaged the data over three trials.

#### 2.3.2. Balance Analysis With Integrated VR

**Figure 2:**
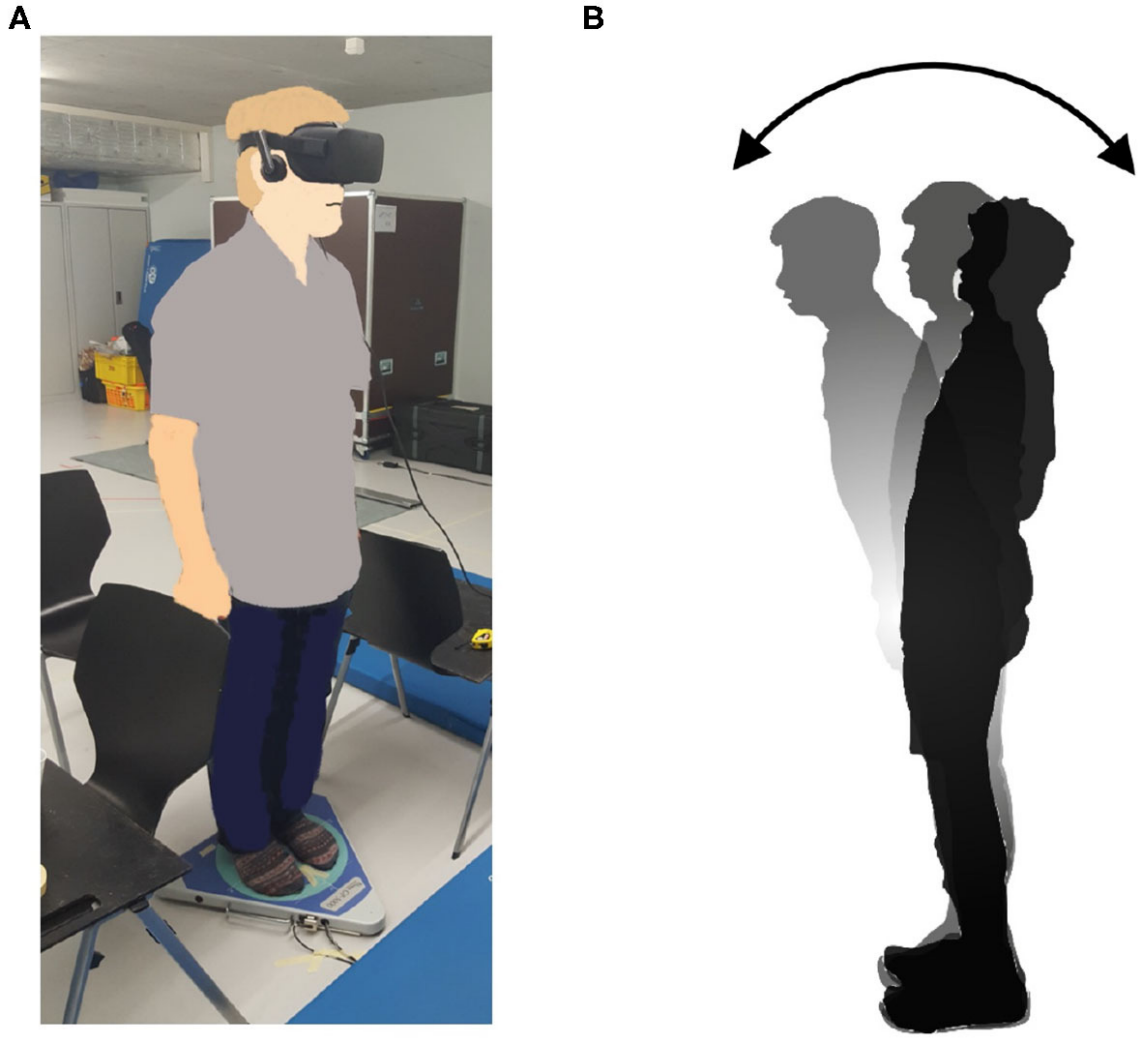
**(A)** New posture assessment system consisting of stabilometer GP-5000 (ANIMA Corp.) and VR headset with HMD Oculus Rift (Oculus VR, LLC). **(B)** Experiment to evaluate whether the intended movements toward forward and backward directions in VR environments stimulate more body sway in the AP direction in the real world.

**Figure 3:**
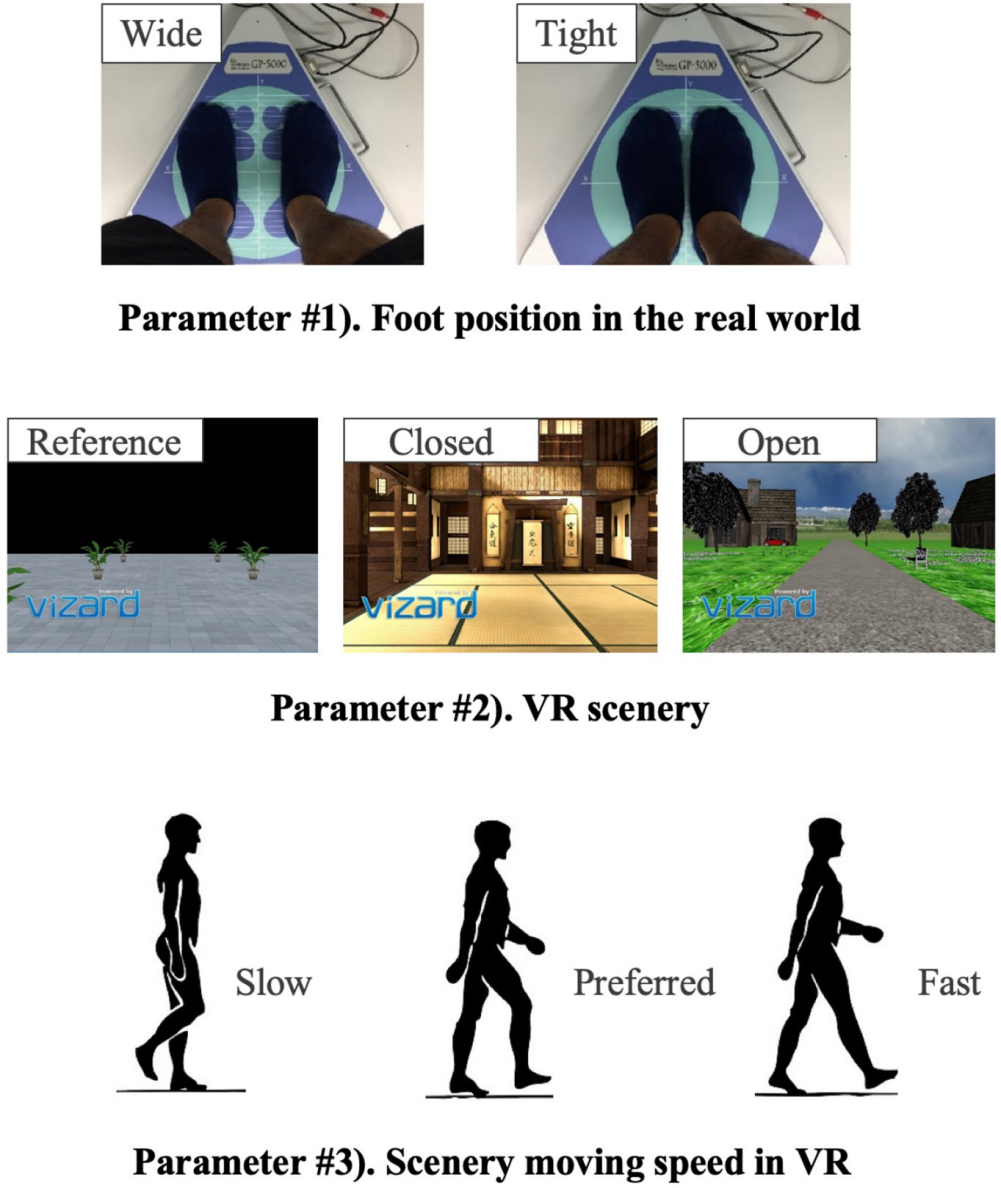
Parameters changed during the balance assessment with integrated VR.

**Figure 4:**
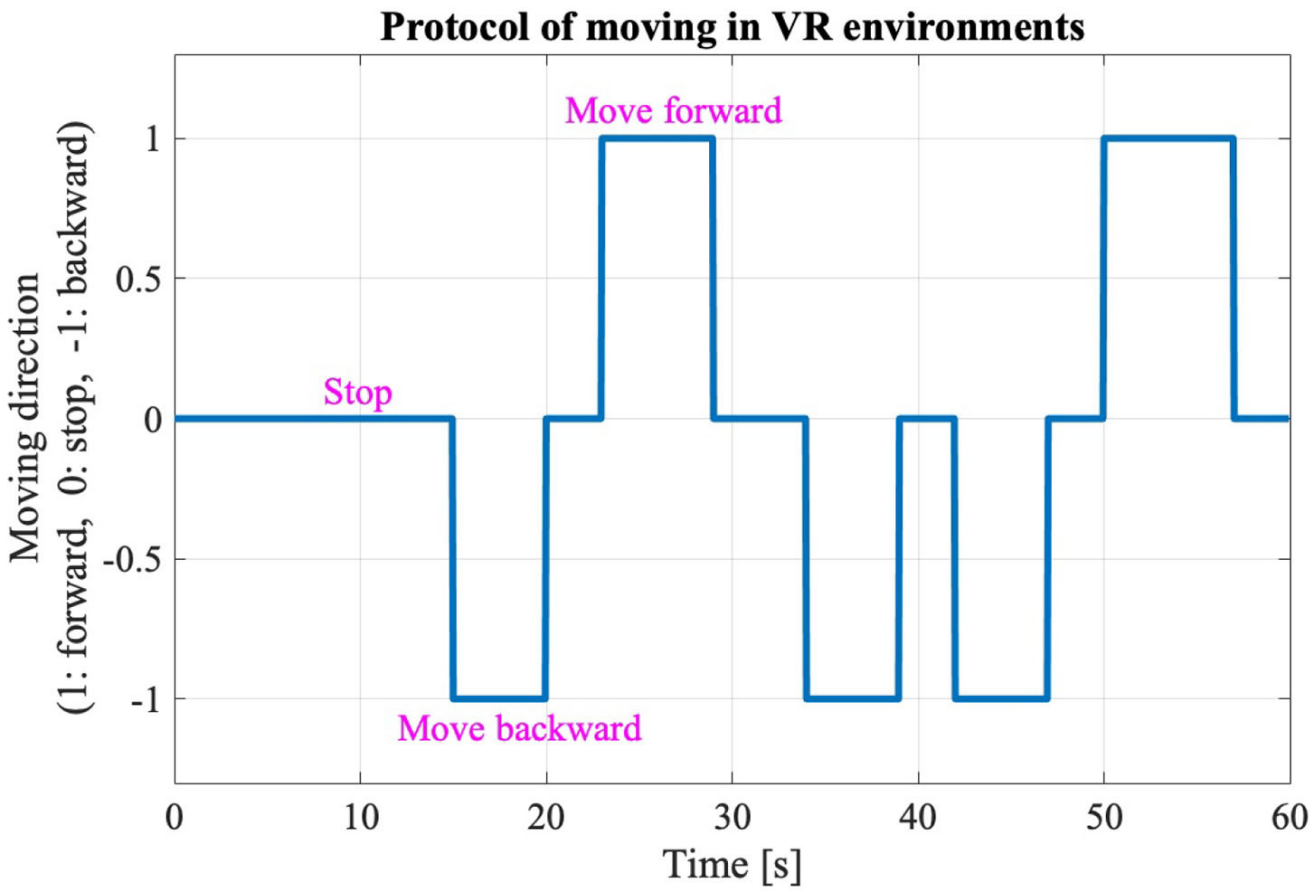
Changes of moving directions in the VR environments.

The new body balance assessment system consists of two main instruments: a stabilometer GP-5000 (ANIMA Corporation, Tokyo, Japan) and HMD-based VR Oculus Rift (Oculus VR, CA, U.S.). Figure 2 illustrates the actual system. We prepared 22 different test conditions by changing three parameters, (1) foot position in the real world, (2) VR scenery, and (3) moving speed of the scenery in VR environments, because the foot position influenced standing balance significantly and the effect of VR immersion was dependent on the complexity of visual scenes displayed in the VR headset (25, 26). More specifically, we arranged (1) two foot positions: tight and wide, (2) three VR sceneries: reference, closed, and open environments (refer to the video files of VR designs in the Supplementary Material), and (3) three moving speeds: slow, preferred, and fast, as illustrated in Figure 3. We set the average individual gait speed, which was measured in the prior gait analysis, as the preferred moving speed in the VR environments. The fast moving speed was calculated based on previous research that measured comfortable and maximum walking speeds of healthy adults aged between 20 and 79 years old (27). From the study, we derived an increase rate of gait speed of 49.25 and 37.50% for older male and female adults, respectively and 81.84 and 75.34% for young male and female samples, respectively. The rate was applied to the individual preferred gait speed to calculate the fast moving speed. The slow moving speed was also derived with the same rate. We intentionally designed the VR environments to move in forward and backward directions with each calculated speed, following the timeline as explained in Figure 4. Finally, to provide different levels of visual stimuli in VR environments, we designed a simple reference scenery with less distraction and closed and open sceneries with more distraction. The VR designs were developed on Vizard VR software (WorldViz, CA, U.S.).

Table 1 shows the detailed combinations of the three parameters in each of the 22 test conditions. Test #1 is the baseline condition and the participants stood on the stabilometer without the VR headset and with eyes-open. Test #2 is equal to test #1, and the participants changed their foot position from tight to wide in test #3. VR environments were integrated in the rest of the tests, from #4 to #22. All the participants started in order from test #1 to #4. Then, the measurement was conducted in the reference VR scenery, while the order between #5 and #10 was randomized. Finally, we performed the rest of the test conditions, from #11 to #22, while randomizing the order partially; we tested three conditions with the same VR scenery first and then changed to the other VR scenery for the next three tests (i.e., three conditions in closed or open VR environment → next three conditions in open or closed environment). As explained in Table 1, the order of small test blocks of 3a–3d was randomized, and the order of the tests in each test block was also randomized. We did not completely randomize the order of the test conditions, considering that the complexity of changing various parameters, especially VR sceneries, might cause safety-related problems such as falling. During the experiment, the participants were asked to stand upright on the stabilometer, with their feet parallel and their arms fixed on each side, and look forward for 60 s in each test. We checked in each test condition whether the participants changed their foot position precisely and put landmarks with tapes for the participants to understand where to stand. The displacement of the Center of pressure (CoP) was measured in both the ML and AP directions by the stabilometer and the data were sampled at 20Hz after being filtered.

**Table 1:** Experiment conditions of balance assessment with VR (* The order of small test blocks from 3a to 3d is randomized).

**Test #**	**Foot position**	**VR scenery**	**Moving speed**	**Order**
#1	Tight	None	None	1. Fixed
#2	Tight	None	None	
#3	Wide	None	None	
#4	Wide	Reference	None	
#5	Wide	Reference	Preferred	2. Randomized
#6	Wide	Reference	Fast	
#7	Wide	Reference	Slow	
#8	Tight	Reference	Preferred	
#9	Tight	Reference	Fast	
#10	Tight	Reference	Slow	
#11	Wide	Closed	Preferred	3a. Randomized
#12	Wide	Closed	Fast	
#13	Wide	Closed	Slow	
#14	Tight	Closed	Preferred	3b. Randomized
#15	Tight	Closed	Fast	
#16	Tight	Closed	Slow	
#17	Wide	Open	Preferred	3c. Randomized
#18	Wide	Open	Fast	
#19	Wide	Open	Slow	
#20	Tight	Open	Preferred	3d. Randomized
#21	Tight	Open	Fast	
#22	Tight	Open	Slow	

### 2.4. Signal Processing and Statistical Analysis

#### 2.4.1. Calculation of Postural Sway Velocity

The raw data of gait and balance were processed on MATLAB R2017b (MathWorks, MA, U.S.). We focused on analyzing velocity of postural sway because a meta-analysis found that significant differences were observed in sway velocity for an MCI group (13). In addition, a few studies developing a similar assessment system showed that sway speed was a sensitive dependent variable (28, 29). We calculated the mean body sway velocity (*MV*) over 50 s after removing the initial 10 s of data to eliminate unusual movements caused by eye focus adjustment in the VR headset. The *MV* was calculated for each participant in each test condition, following formulae (1) and (2), to investigate the differences between the test conditions and between the populations. We also calculated the postural sway velocity averaged over each group (OA and YA) in both the AP and ML directions in each test condition to evaluate the differences. *N* is the number of samples, *AP*_*i*_ and *ML*_*i*_ are the displacement of CoP in the AP and ML directions, respectively, at *i*th sample, and *T* is the sampling interval.

(1) $$\begin{array}{r} MV_{AP}=\frac{1}{N-1}\overset{N-1}{\underset{i=1}{\sum }}\frac{\left|AP_{i+1}-AP_{i}\right|}{T} \end{array}$$

(2) $$\begin{array}{r} MV_{ML}=\frac{1}{N-1}\overset{N-1}{\underset{i=1}{\sum }}\frac{\left|ML_{i+1}-ML_{i}\right|}{T} \end{array}$$

#### 2.4.2. Effect of the VR Environments on Postural Behavior in the Real World

The mean postural sway velocity, *MV*_*AP*_ and *MV*_*ML*_, of each participant in each test condition was analyzed statistically on R version 3.6.0. We calculated the *P*-values (5% significance level) and effect size, by comparing each of the *MV*_*AP*_ and *MV*_*ML*_ between baseline condition (#1) and each of the others (#2 to #22) in each participant to investigate the effect of VR scenarios on sway movements in the real world. We evaluated the data distribution and homogeneity of variance and thus used the paired Wilcoxon signed-rank test. In addition, we inspected the effect by analyzing the sway velocity data on frequency domain. We performed a Fast Fourier Transform (FFT) to the velocity data of 50 s in each test condition for each participant. We then applied Welch's method to the transformed data to estimate the power spectral density (PSD) and re-sampled the calculated PSD data into 0.04Hz bins. Subsequently, we calculated the absolute difference in the re-sampled PSD of each frequency bin between the baseline #1 condition and each of the other test conditions for each participant. We then averaged the difference of PSD over each group (OA and YA).

#### 2.4.3. Evaluation of the Difference Between Groups (OA and YA)

We compared the mean sway velocity data of *MV*_*AP*_ and *MV*_*ML*_ between OA and YA in each test condition to examine the differences. We performed the unpaired Wilcoxon signed-rank test to compare each of *MV*_*AP*_ and *MV*_*ML*_ between OA and YA in each test condition.

#### 2.4.4. Effect of the Changes of Foot Position and VR Environments

After evaluating our primary hypotheses, we further explored the measured data. First, we examined the impact of foot position on body balance, using the “nparLD” function for non-parametric analysis of variance (ANOVA) because of the data distribution (30). We performed the non-parametric ANOVA, focusing on the test conditions #5–10 due to the non-randomization between VR sceneries as discussed above. We supposed that there could be a learning effect due to the partial randomization of test conditions between the three VR sceneries if we included all the test conditions in the analysis. Second, we investigated the effect of moving speeds of the VR scenery, focusing on the test conditions #4–7 with the reference VR scenery, for the same reason of non-randomization. We performed the non-parametric ANOVA by comparing the test condition #4 and each of the others #5–7.

#### 2.4.5. Association Between Postural Sway and Cognitive Function

Focusing on the postural sway in the AP direction, where we intentionally triggered the movement, we inspected the association between the MoCA score and mean body sway velocity, *MV*_*AP*_, based on the assumption that there could be relationship between cognitive skill and postural movements. We performed Spearman rank-order correlation analysis for each population group.

## 3. Results

### 3.1. Participants

A total of 14 healthy OA and 16 healthy YA joined the experiment, though one young adult dropped out due to a motion sickness during the balance assessment with reference VR scenery. Significant differences at 5% level were observed in age, BMI, and MoCA between the two groups as shown in Table 2.

**Table 2:** Demographic profile of participants.

**Variable**	**OA**	**YA**	***P*-value**
Gender	Men: 6, Women: 8	Men: 7, Women: 9(1 dropped out)	
Age (years)	73.14 ± 4.26	24.93 ± 1.49	*P* < 0.001
Weight (kg)	77.26 ± 19.39	68.73 ± 14.64	*P* = 0.201
Height (cm)	168.43 ± 7.91	171.80 ± 9.28	*P* = 0.444
BMI	27.00 ± 5.21	23.07 ± 3.37	*P* = 0.012
MoCA score	27.79 ± 1.89	29.27 ± 1.03	*P* = 0.029
Gait speed (cm/s)	126.66 ± 15.06	137.68 ± 22.82	*P* = 0.354
Stride length (cm)	129.53 ± 14.99	141.94 ± 16.59	*P* = 0.061

### 3.2. Mean Velocity of Postural Sway

**Figure 5:**
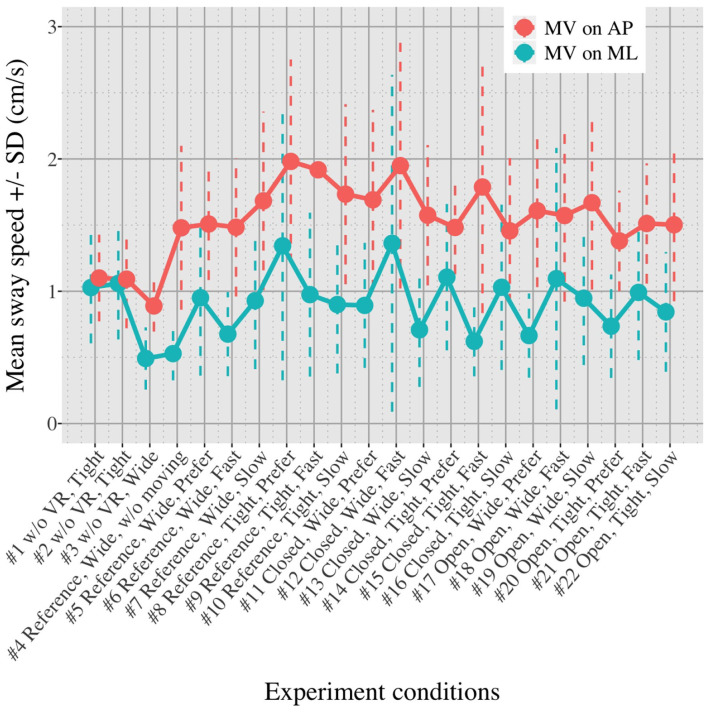
Average *MV*_*AP*_ and *MV*_*ML*_ in healthy OA in each test condition.

**Figure 6:**
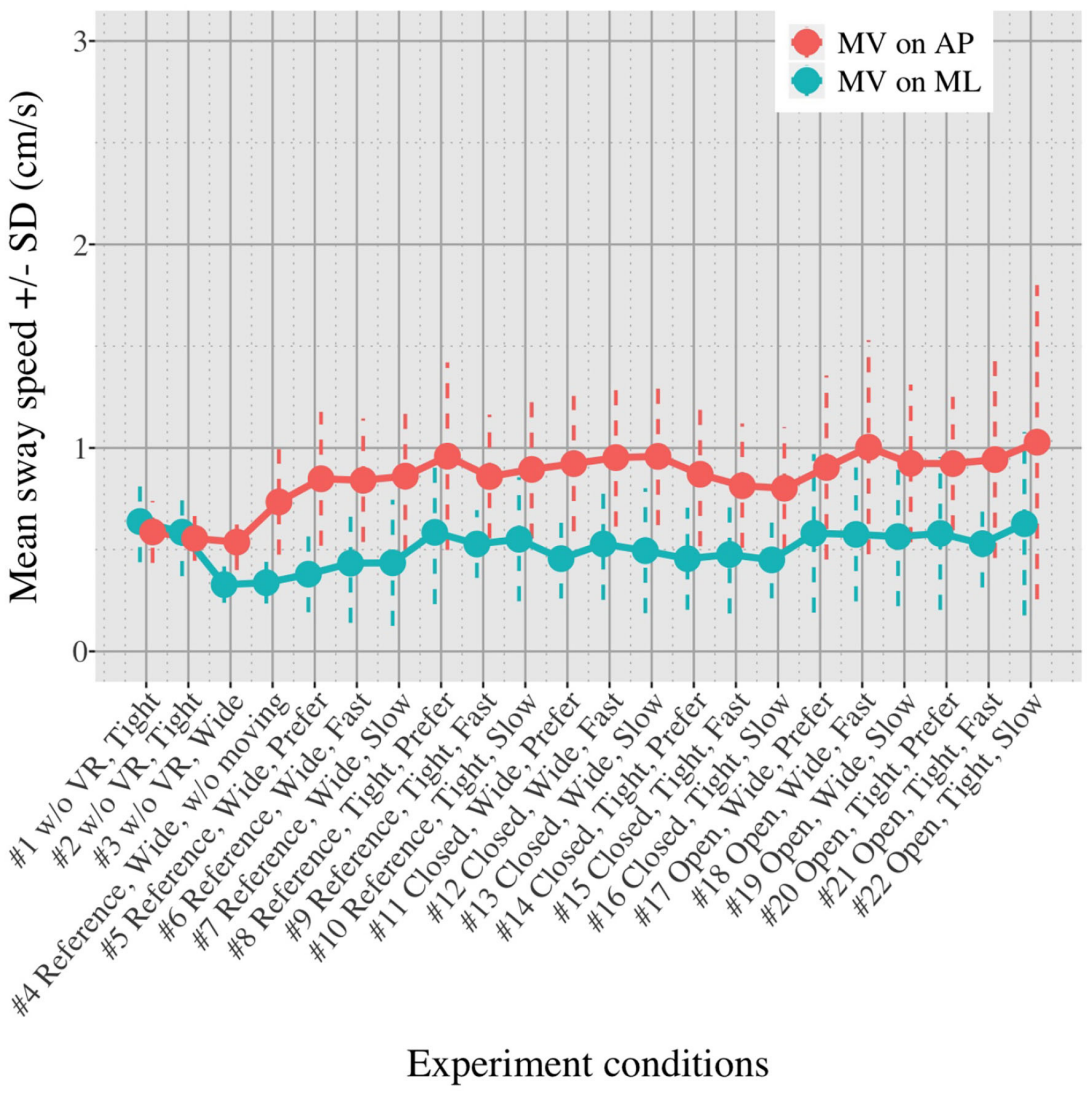
Average *MV*_*AP*_ and *MV*_*ML*_ in healthy YA in each test condition.

Figures 5, 6 show the mean *MV*_*AP*_ and *MV*_*ML*_ averaged over each group (OA and YA) in each of the 22 test conditions.

### 3.3. Comparison of Postural Sway Velocity Between Baseline Condition and Each of the Other Conditions

**Figure 7:**
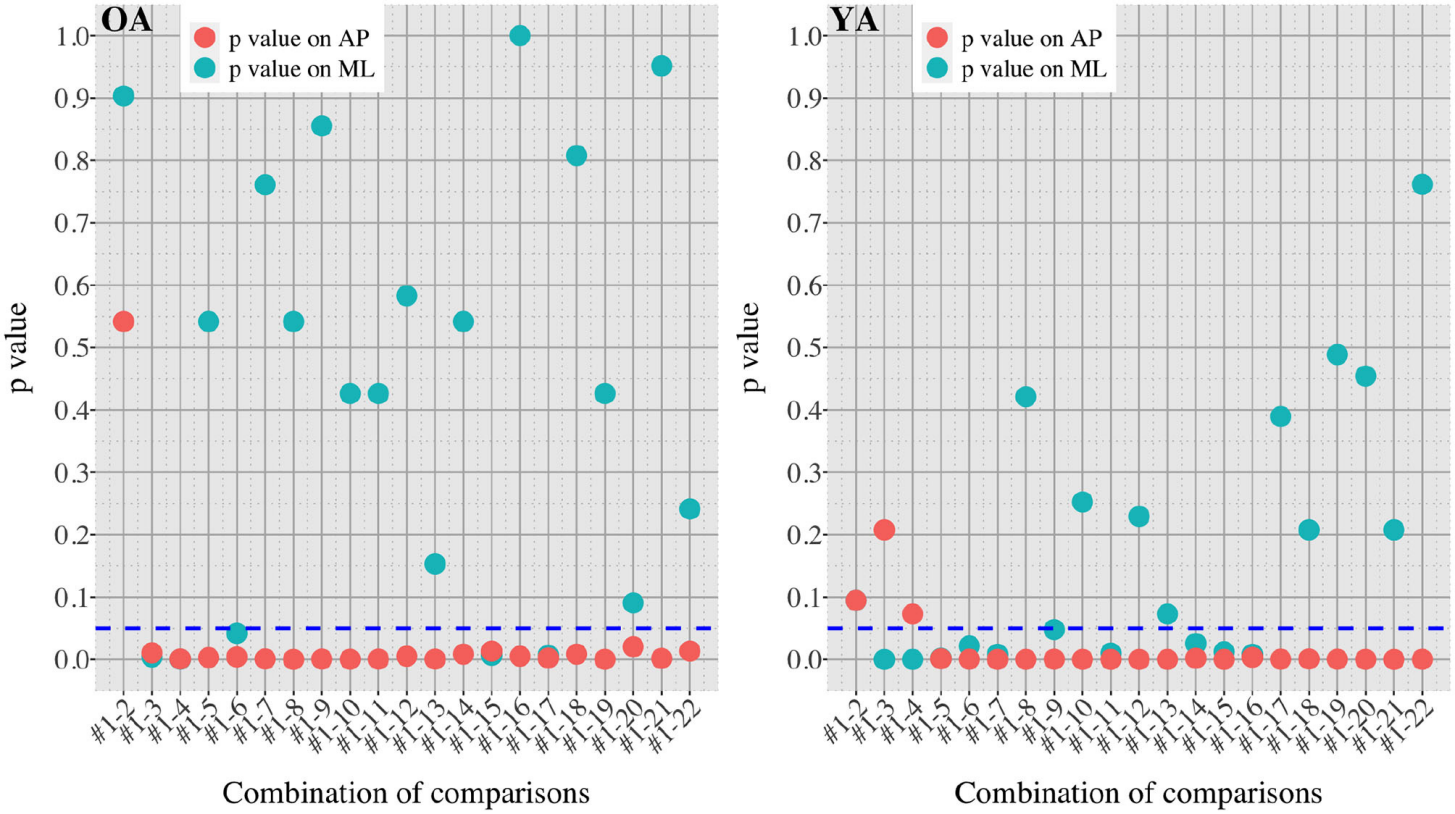
*P*-values in all the comparisons between baseline condition #1 and each of the other conditions in healthy OA and YA.

**Figure 8:**
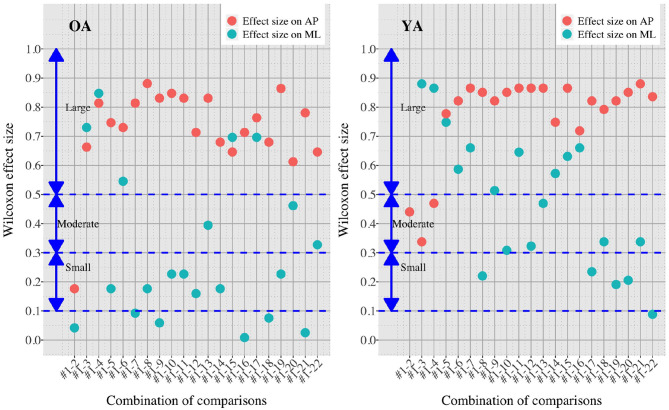
Effect sizes in all the comparisons between baseline condition #1 and each of the other conditions in healthy OA and YA.

**Figure 9:**
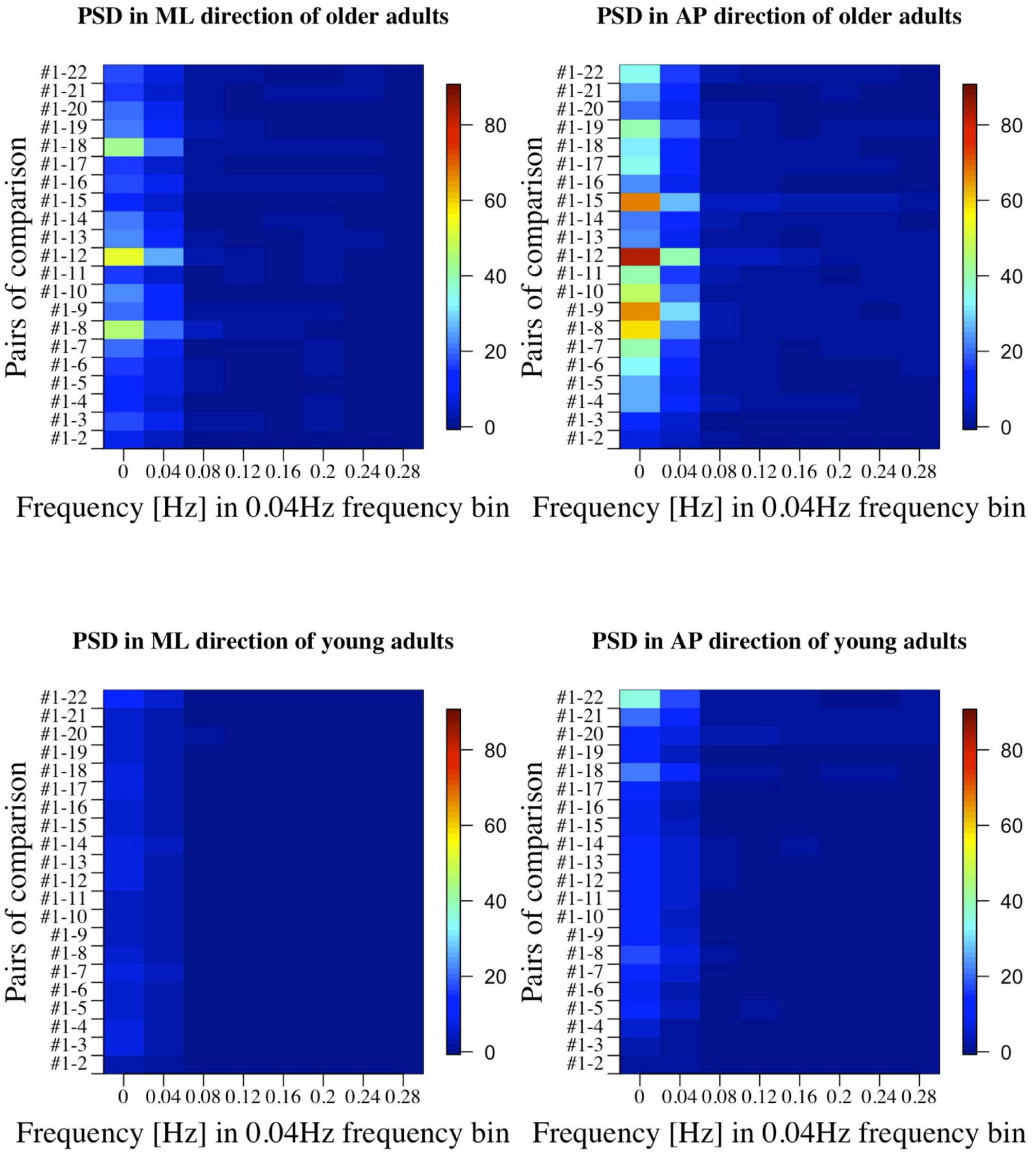
PSD in all the comparisons between baseline condition #1 and each of the other conditions in healthy OA and YA.

Figures 7, 8 show the results of comparisons between baseline condition #1 and each of the other conditions in terms of *P*-value and effect size. In addition, the differences in PSD of sway velocity between baseline condition #1 and each of the others is illustrated in Figure 9, focusing on the frequency range from 0 to 0.28 Hz.

### 3.4. Comparison of Postural Sway Velocity Between Healthy Older and Young Adults

**Figure 10:**
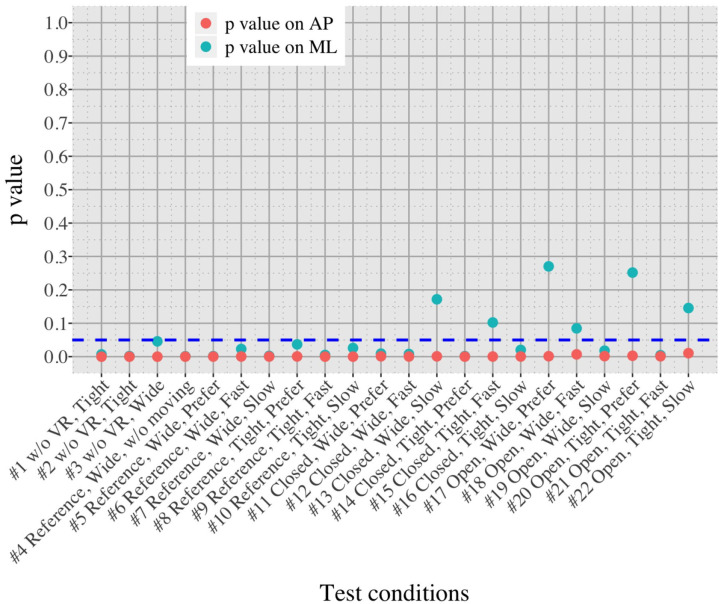
Comparison of postural sway velocity between OA and YA in each of the 22 test conditions.

The differences in postural sway between OA and YA is illustrated with *P*-values in each of 22 test conditions (Figure 10).

### 3.5. Comparison of Postural Sway Velocity Between the Test Conditions With Reference VR Scenery

#### 3.5.1. Effect of Foot Position

**Figure 11:**
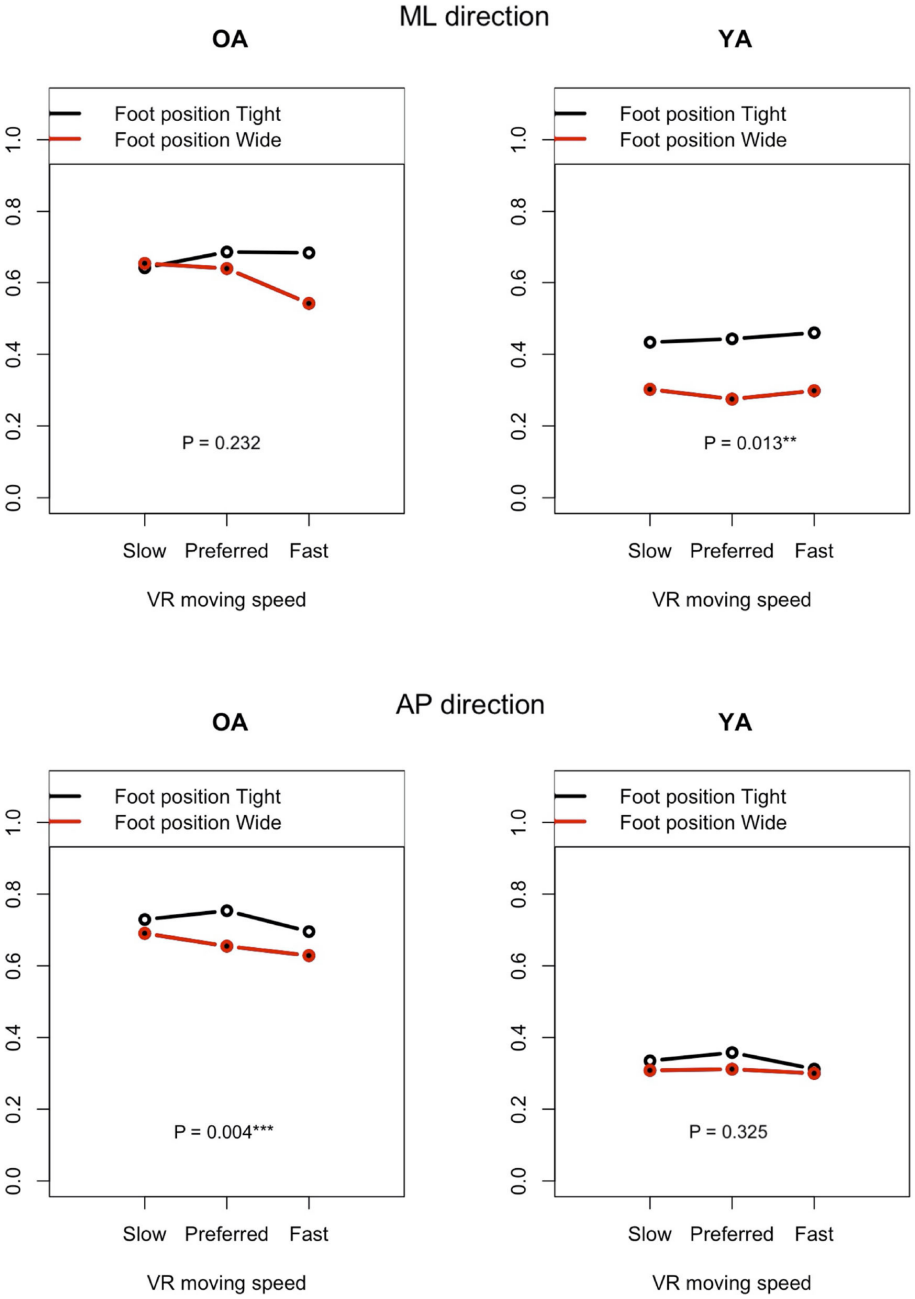
Effect of foot position on postural sway velocity in each of the ML and AP directions, reference VR scenery (vertical axis: normalized relative effect).

Figure 11 shows the impact of foot position in each group (OA and YA) in both the ML and AP directions, comparing the mean sway velocity between the test conditions #5–10 with using a normalized relative effect.

#### 3.5.2. Effect of Moving Speeds in the VR Environments

**Figure 12:**
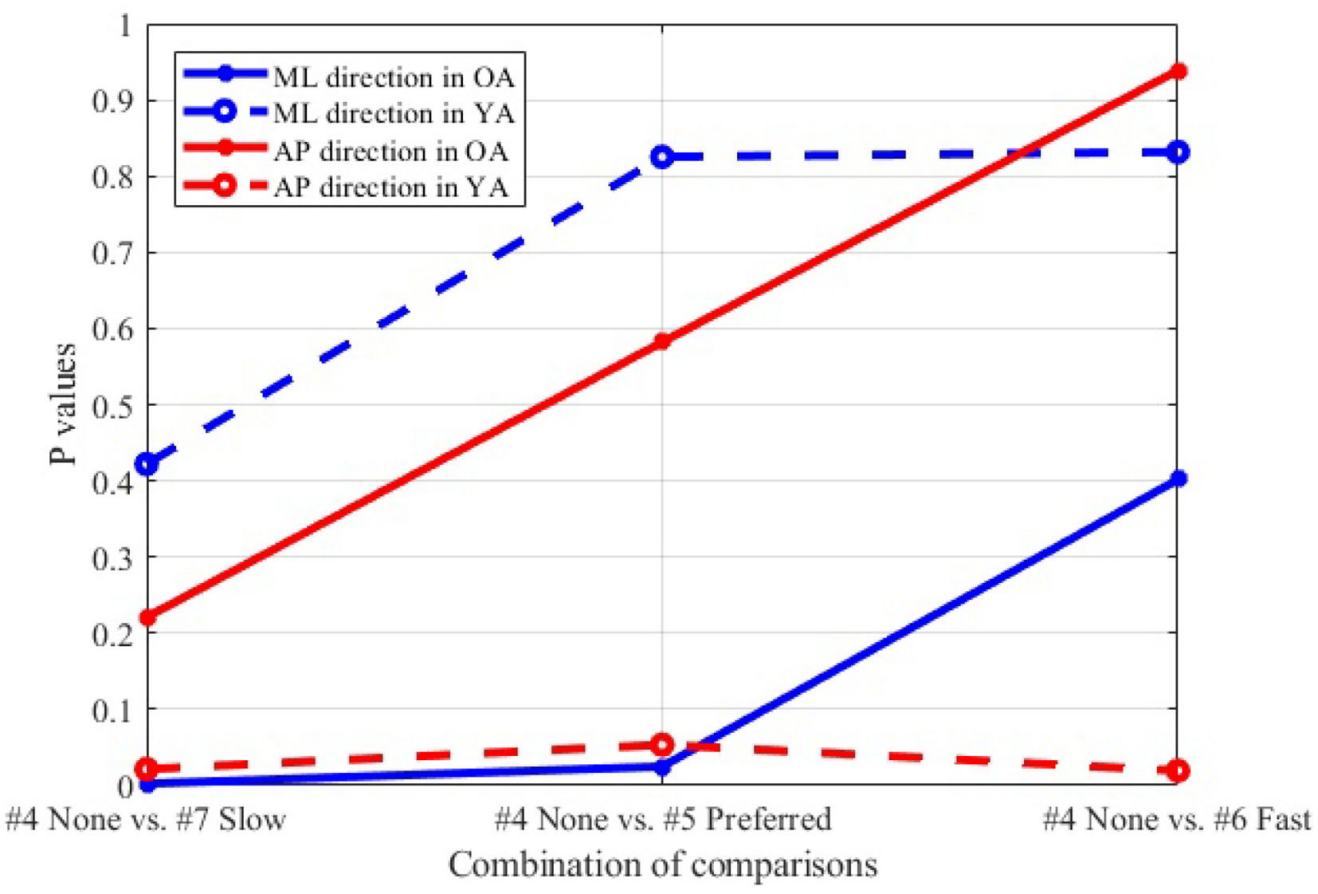
Comparison between non-moving (#4) and each of three different speeds (#5–7), wide stance, reference VR scenery.

Figure 12 shows the *P*-values of each comparison between the non-moving VR environment (#4) and each of the other three conditions with the moving VR environments (#5–7) in both the ML and AP directions.

### 3.6. Correlation Between Mean Postural Sway Velocity in the AP Direction and MoCA Score

**Figure 13:**
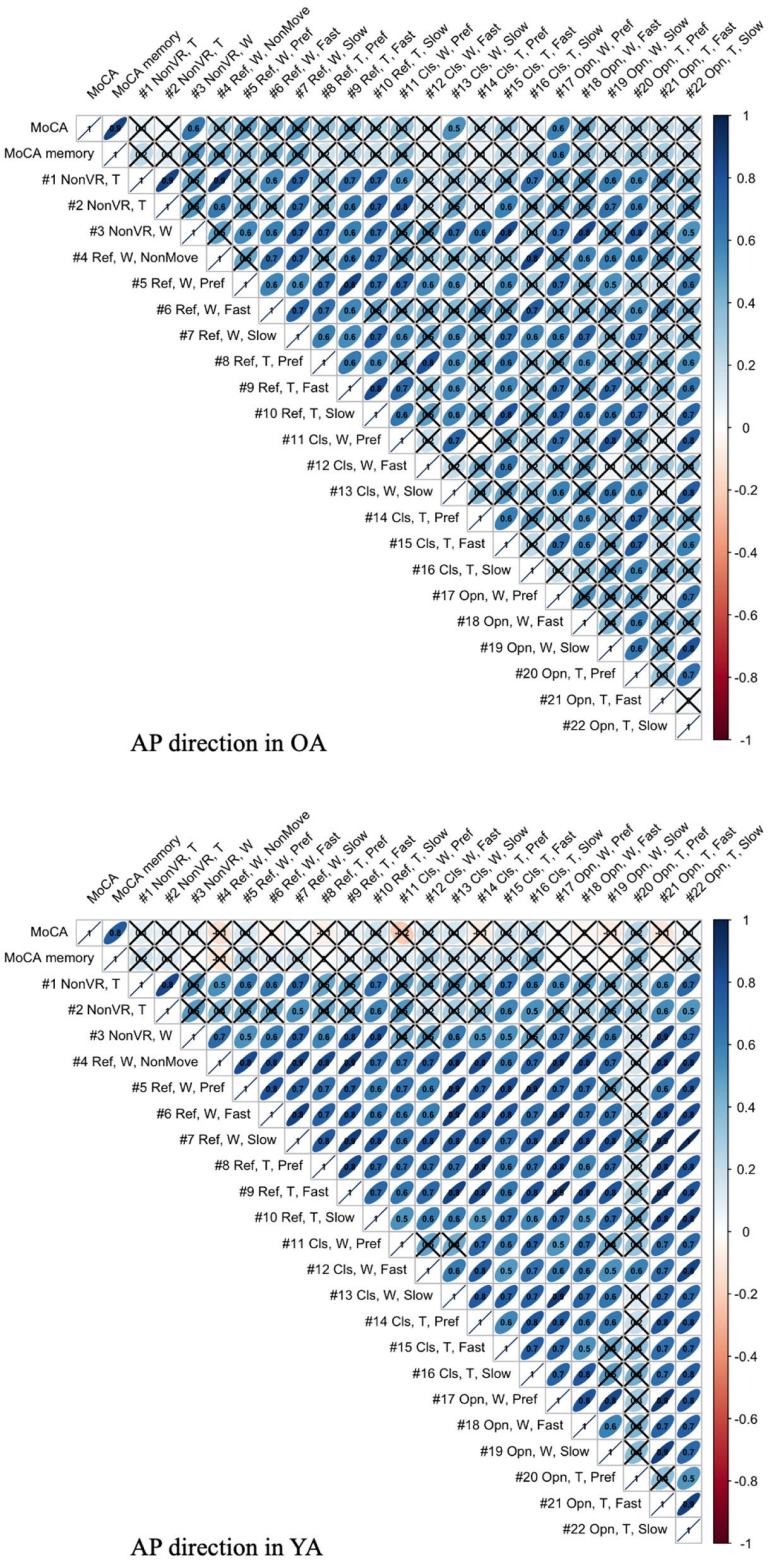
Association between MoCA score and mean body sway velocity in the AP direction of each test condition for YA and OA (T, Tight foot position; W, Wide foot position; Ref, Reference VR scenery; Cls, Closed VR scenery; Opn, Open VR scenery; Pref, Preferred moving speed).

The association between mean velocity of body sway in the AP direction and MoCA score is illustrated in Figure 13. The figure visualizes the correlation values, with × meaning that the *P*-values of correlation is above the significance level of 5% and, thus, the association shows lower significance.

## 4. Discussion

### 4.1. Effect of Imposed Movements in VR Environments on Postural Sway in the Real World

Figures 5, 6 reveal that both OA and YA swayed more in the AP direction under the VR environments, whereas they swayed with a similar velocity in the ML direction across all the conditions. The velocity in the AP direction is increased by 1.5 to 2 times from the baseline for both groups. Interestingly, the average velocity fluctuates less in YA than in OA throughout all the test conditions. As shown in Figure 7, the Wilcoxon signed-rank test suggests that significant differences are seen in the AP direction for all the comparisons except one (#1–2) in OA and three (#1–2, #1–3, and #1–4) in YA (OA: *P* < 0.020* and YA: *P* < 0.003*). On the other hand, five comparisons in OA and 10 comparisons in YA show significant differences in the ML direction. Moreover, the effect size (Figure 8) shows that the size in the AP direction is large in all the comparisons except one (#1–2) in OA and three (#1–2, #1–3, and #1–22) in YA (OA: effect size> 0.61 and YA: effect size> 0.72). Less significant differences and small or moderate effect size are seen in the comparison of #1–2 for both groups, which as expected since these conditions are the same. In contrast to the AP direction, the effect sizes in the ML direction tend to show large variability. Similarly, the PSD analysis in Figure 9 visually indicates that the postural sway of both OA and YA is affected more in the AP direction under VR environments, particularly in the frequency range from 0 to 0.04Hz.

Overall, the results show that both groups swayed significantly in the AP direction in the real world, being affected by the movements in forward and backward directions in the VR environments, as hypothesized. This may indicate that our new body balance assessment system with HMD-based VR technology can improve the sensitivity of posture analysis compared to conventional methods. We found significant differences between eyes-open baseline condition #1 and the conditions with VR environments in OA, while a conventional balance assessment observed no significant differences between eyes-open (equal to our test #1) and eyes-closed conditions in the elderly (31). Interestingly, our results are in line with the findings of the few previous studies that also evaluated similar combined systems. In particular, CoP and ellipse area were measured for healthy YA under the conditions of eyes-open without VR and two different VR sceneries: open and closed environment (28). The result showed significant differences in the sway velocity between with and without VR environments and between open and closed VR sceneries. Similarly, HMD-based VR was used to generate visual stimuli and the effect of stimuli on postural control was evaluated under the conditions of eyes-open, eyes-closed, and three different VR designs; e.g., an optokinetic drum rotating around yaw, pitch, and roll axes, respectively (29). This research also reported differences between with and without VR environments in postural sway behavior of healthy YA.

### 4.2. Differences Between Healthy Older and Young Adults

We initially assumed that the differences between the groups were smaller in the conditions without VR environments. However, significant differences were observed for all the test conditions in the AP direction (*P* < 0.010) and for 16 tests in the ML direction (*P* < 0.046) (Figure 10) between OA and YA. The result reveals that the mean sway velocity was different significantly between the groups in even the baseline condition #1 and that the VR environments we designed might not be enough to trigger a larger difference between the groups. Nonetheless, it may be also important to consider the effect of foot position in the real world. As shown in Figure 5, less variability of the mean sway velocity and a slower mean sway speed are observed among the OA group for the condition #3 with wide foot stance and without a VR environment, in comparison to the baseline condition #1. The standing with wide foot position might have enabled OA to stand more stably, canceling other aging effects (e.g., weak lower-limb muscle strength) on their musculoskeletal function especially in the ML direction and leading to the less variability and decreasing mean sway velocity. Interestingly, the difference between OA and YA is nearly not significant in the ML direction for the test condition #3 (*P* = 0.046, Figure 10). Therefore, the difference in the baseline condition #1 could be due to a decline of motor function. It may be important to eliminate aging factors related to motor functions to inspect the effect of VR environments between the groups more precisely.

On the other hand, when the significance level is set to 1% in Figure 10, twenty one tests in the AP direction and ten tests in the ML direction show significant differences (*P* < 0.007 for AP, *P* < 0.009 for ML). In particular, assuming that wide foot position could lead to more precise evaluation of the effect of VR environments as described above and thus focusing on the test conditions with wide foot position, the test conditions #4, #5, #7, #11, and #12 show the significant differences in both the AP and ML directions between the groups at 1% significance level. Hence these five test conditions could distinguish the two groups more clearly. In addition, the effect sizes in Figure 8 also explain an interesting difference between the groups. Overall, the effect sizes are larger for YA than OA in both the AP and ML directions. When we set a new threshold at 0.8 and divide the large effect size into two, the effect sizes of the comparison between #1 and #12 come into small (ML) and large< 0.8 (AP) for OA, and moderate (ML) and large> 0.8 (AP) for YA. Therefore, the evaluation of relative changes between the specific test conditions could improve the classification between the groups.

To summarize, the results suggest that differences in the AP direction are possibly a better indicator to distinguish between populations. Previous research also found age-related differences in a conventional body balance assessment for both eyes-open and eyes-closed conditions for mean velocity in the AP direction (31). In addition, the classification between the groups could be improved if we remove the factors of motor functions and integrate the evaluation of relative changes of sway velocity. However, sway parameters in the ML direction could be also helpful for the classification. YA seems to be more sensitive to movements in the ML direction than OA especially because the *P*-values are smaller and the effect sizes are larger under the VR environments compared to OA (Figures 7, 8). Further analysis of postural sway in the ML direction could lead to another relevant discriminator between the groups.

### 4.3. Impact of Foot Position and Moving Speeds With Reference VR Scenery

#### 4.3.1. Foot Position

The resulting analysis (Figure 11) shows a significant difference for YA between the foot positions in the ML direction (*P* = 0.013), while no significant difference is seen in the AP direction (*P* = 0.325). This result may indicate that YA swayed less in the ML direction and swayed similarly in the AP direction when they changed the foot position from tight to wide because they were probably able to maintain their posture more stably in the ML direction with wide foot position, as expected. On the other hand, no similar difference is found in OA. Contrary to the result of YA, a significant difference is observed in the AP direction but not in the ML direction. Since we checked whether the participants changed their foot position precisely during the experiment, the finding will not be due to the wrong foot position. A notable point is that the difference becomes larger in the ML direction between the foot positions as the moving speed of VR scenery increases. This may suggest that OA stabilized their posture in the ML direction, paying more attention to the increasing speed of visual flow moving forward and backward in the VR environments. However, motor function could still affect the results. YA might have more muscle strength to adjust to the changes of foot position more easily compared to OA.

#### 4.3.2. Moving Visual Flow in Reference VR Scenery

The result (Figure 12) indicates that, particularly in OA, more significant postural sway velocity is observed as the moving speed decreases in the VR environment. Remarkably, the result is in agreement with previous studies that found a decreased postural stability in the case of slower real world walking (32, 33). This finding hints to the fact that the VR environment created in this study induces a sense of visual flow comparable to the experience that we feel in the real world while walking.

### 4.4. Association Between Postural Sway Behavior and Cognitive Skill

As shown in Figure 13, the meaningful association is not seen in YA between MoCA and all the test conditions, as expected, since 60% of YA reach the maximum score in MoCA, possibly indicating a ceiling effect. On the other hand, the result shows the relevant association between MoCA and the test conditions, #3, 13, and 17, in OA. Interestingly, the common parameter in these conditions is wide foot position. The result might suggest that wide foot position enables the newly developed balance assessment system to detect the postural movements related to cognitive functions by eliminating the noisy postural movements caused by potentially weak lower-limb muscle strength. However, we should consider that the same trend is not observed in the other test conditions with wide foot position and that the sample size is small.

### 4.5. Limitations and Future Work

While the new body balance assessment system with integrated VR technology gives interesting outcomes, we acknowledge the limitations of our study.

First, we did not randomize the order of the test conditions completely due to safety concerns. The incomplete randomization could have led to a learning effect of the participants, affecting their body sway. In fact, the OA might have got used to the VR environments because the mean velocity of postural sway in the AP direction slightly decreased after they completed the first 10 tests (Figure 5). However, the same tendency is not observed in YA. Considering that all the participants, except for one young adult, had never experienced HMD-based VR technology before this measurement, OA might be more prone to be affected by the changing parameters in comparison to YA, causing the decreasing velocity in the AP direction seen in the latter half of the test conditions.

Second, we did not consider the impact of the weight and wearing of the VR headset. Specifically, two different elements were added between the tests #3 and #4; the participants wore the VR headset and saw the reference VR scenery. We should have prepared another test condition to evaluate the effect of the weight and wearing without showing any VR designs. This could help us understand whether the large effect size in the AP direction seen in the comparison of #1 and #4 (Figure 8) is due to the weight and wearing, the implementation of reference VR scenery, or the combinations of these factors. This meticulous classification will be important since OA swayed significantly in the AP direction even when they just changed their foot position from tight to wide without VR environments (see the comparisons of #1 and #2, and #1 and #3 in Figure 7). Therefore if we eliminate the potential noisy elements (e.g., weight and wearing of VR headset, lower-limb muscle strength), our analysis could result in more detailed and precise values for OA, particularly considering that there are significant differences in PSD of body balance even between different groups of healthy elderly adults (34).

Third, further enhancements of data analysis would provide more meaningful outcomes for us to establish a more reliable balance assessment system for diagnosis of dementia in the future. We analyzed the data in both time and frequency domains in this study. While we find similar general results in both analyses, differences are also seen in specific points. For example, while the comparison of #1-12 shows the largest PSD in OA (Figure 9), the effect size is not the largest, as shown in Figure 8. Therefore, more relevant variables to the diagnosis of dementia could be discovered if we integrate more various parameters into our analysis as discussed in (35).

Finally, while VR technology is likely to have the potential to improve both assessments and interventions, it is not yet always available in a clinically reasonable state of development. Further validation studies and technological improvements are necessary to deliver the technology to clinically applicable settings.

## 5. Conclusion

The new balance assessment system with integrated VR environment stimulated body sway in the AP direction for both OA and YA as hypothesized. Moreover, we confirmed a more significant velocity of postural sway in OA. The presence of AP displacement could be an early sign of postural control impairment in people threatened to develop dementia and the newly developed balance assessment system with HMD-based VR technology might be useful in detecting small postural instabilities in the preclinical stage of dementia. To establish the new assessment system as a diagnosis tool for dementia in the future, we will improve the research design as discussed above and conduct additional measurements with people suffering from dementia to understand more specific and relevant parameters to diagnosis. The result of this study warrants a cross-sectional study in which OA diagnosed with and without dementia are compared in terms of their sway behavior.

## Data Availability Statement

The datasets generated for this study are available on request to the corresponding author.

## Ethics Statement

The studies involving human participants were reviewed and approved by ETH Zurich Ethics Commission. The patients/participants provided their written informed consent to participate in this study. Written informed consent was obtained from the participant in the figures for the publication of any potentially identifiable images or data included in this article.

## Author Contributions

Each of the authors has contributed to developing the research concept and experimental designs. YI and NS performed the measurement, first analysis, and interpretation of the data. In addition, all authors contributed to drafting and revising the article to bring it to its current state. All authors approved the final version to be published.

## Conflict of Interest

The authors declare that the research was conducted in the absence of any commercial or financial relationships that could be construed as a potential conflict of interest.
