# Supplementary material for: Triggering Postural Movements With Virtual Reality Technology in Healthy Young and Older Adults: A Cross-Sectional Validation Study for Early Dementia Screening
Source: Front Med (Lausanne). 2020 Nov 16;7:533675. doi: 10.3389/fmed.2020.533675 (PMC7701173; doi:10.3389/fmed.2020.533675)
Supplement: Supplementary file 4 [file Data_Sheet_1.pdf]

Supplementary Material

1 VIDEO FILES OF VIRTUAL REALITY SCENERY

We developed virtual reality (VR) scenery on Vizard VR software (WorldViz, CA, U.S.). The following video files show the actual VR scenery that the participants watched in the VR environments during the experiment.

- `Video_1_Reference_VR_scenery.avi` : Reference VR scenery
- `Video_2_Closed_VR_scenery.avi` : Closed VR scenery
- `Video_3_Open_VR_scenery.avi` : Open VR scenery
